# Innovative Management of Aerophagia-Induced Intestinal Pseudo-Obstruction in an Adult With Severe Intellectual Disability: A Case Report

**DOI:** 10.7759/cureus.78170

**Published:** 2025-01-29

**Authors:** Shoko M Yamada, Shozo Terada, Takane Harada, Yoshio Nehashi, Noriko Mori

**Affiliations:** 1 Neurosurgery, Teikyo University Hospital, Mizonokuchi, Kawasaki, JPN; 2 Internal Medicine, JCHO Sakuragaoka Hospital, Shizuoka, JPN

**Keywords:** aerophagia, enteral nutrition, intestinal pseudo-obstruction, mental developmental disability, nasogastric tube (ngt)

## Abstract

Intestinal pseudo-obstruction caused by aerophagia is predominantly observed in children with developmental delays, and adult cases are rare. Cases are typically managed symptomatically because there are no known effective treatments. We report here a successful response to intestinal pseudo-obstruction in an adult patient presenting with chronic abdominal distention. A 67-year-old woman with severe intellectual disability was admitted to our hospital with vomiting, fever, and dyspnea. Imaging revealed aspiration pneumonia and significant gas accumulation in the stomach and intestines. During hospitalization, the cause of the intestinal pseudo-obstruction was identified as aerophagia due to frequent and involuntary swallowing of saliva.

Clonazepam and antiparkinsonian drugs were administered to reduce the severity of the salivation, and abdominal massage and intestinal prokinetic agents were used to promote the expulsion of colonic gas. Although these techniques provided symptomatic relief, the underlying aerophagia remained unresolved, and the gastric and intestinal air volume did not improve. After several days of central venous nutrition, the patient was switched to enteral nutrition. We aimed to minimize the passage of air from the stomach into the intestines through a variety of methods, including (1) the use of enteral nutrition that immediately solidified in the stomach, (2) decreased meal frequency to twice daily, (3) placement of a 16 Fr nasogastric tube that was kept open at all times except when enteral nutrition was administered, and (4) positioning of a gastric tube at a depth of 5-10 cm from the gastric cardia so that air stored in the gastric fundus could be easily drained through the nasogastric tube.

These procedures dramatically reduced the gastric and intestinal gas volume, and the abdominal distention resolved. The aspiration pneumonia was successfully treated, and 10 days after the above procedures were implemented, there was no recurrence of gastric and intestinal gas retention, and the patient was discharged for home care. It is not clear whether the procedures utilized in our case would be effective in pediatric patients. However, the approach may be useful for patients with chronic abdominal distension due to aerophagia, especially in home care settings.

## Introduction

Pathologic aerophagia is observed in 8.8% of patients with severe intellectual disabilities [1] and can lead to extensive intestinal gas retention [1-3]. It has been suggested that aerophagia in these patients results from involuntary paroxysmal cricopharyngeal sphincter contractions [4-6], with a high incidence of air swallowing despite normal swallowing frequency [7,8]. Significant abdominal distension from excessive colonic gas accumulation requires differentiation from ileus. When no organic stenosis or obstruction is noted and ileus is ruled out, a diagnosis of intestinal pseudo-obstruction can be made [9]. There is no effective treatment for aerophagia, and most cases are managed symptomatically. Observation is sufficient for asymptomatic patients; however, immediate action is necessary for cases presenting with vomiting, due to the risk of intestinal perforation [10]. Pathological aerophagia is frequent in children but rare in adults [9]. Here, the authors report the successful management of intestinal pseudo-obstruction caused by aerophagia in an adult with severe intellectual disability.

## Case presentation

**Figure 1:**
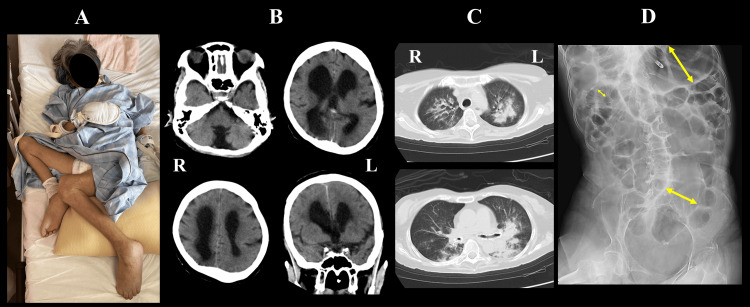
Patient’s condition on admission (A) Both hip and knee joints in the patient were contracted and fixed in a flexed position; (B) Brain CT shows aplasia of the cerebellar vermis and the presence of obviously enlarged ventricles; (C) Chest CT demonstrates evidence of pneumonia in the bilateral lung fields, predominantly in the left lung, with peribronchial and posterior localization suggestive of aspiration; (D) Abdominal X-ray reveals significant intestinal gas retention resembling ileus. The maximum diameters in the stomach, small intestine, and colon, where gas retention was observed, were measured on X-ray (yellow arrows).

A 67-year-old woman was admitted to our hospital with vomiting and high fever (Day 0). The patient had been in a vegetative state, requiring full assistance since childhood due to a severe intellectual disability, and was unable to speak or communicate. Her lower extremities were stiff in a flexed position (Figure 1A), and she occasionally exhibited purposeless upper limb and hand movements. The patient had been on enteral nutrition in home care and demonstrated chronic abdominal distention for the past 10 years. Vomiting occurred every month when the abdominal distention became excessive, but it subsided spontaneously. When the vomiting continued for a few days, which happened four to five times a year, the family gave her abdominal massage, but aspiration pneumonia was never caused by vomiting. A week prior to admission, the patient vomited with a distended abdomen and subsequently developed fever and labored breathing. Her percutaneous oxygen saturation (SpO_2_) dropped to 90%-91% on room air, so she was brought to our hospital by ambulance. At the time of admission, her blood pressure was 141/87 mmHg, heart rate 144 bpm, body temperature 38.0°C, and SpO_2_ was 95% on 5 L/min of oxygen by mask. Brain computed tomography (CT) scan demonstrated aplasia of the cerebellar vermis and marked ventricular enlargement (Figure 1B). Chest CT revealed predominantly left-sided pneumonia; however, the diagnosis of aspiration pneumonia was made because of thickened central bronchi and irregular opacities in the dorsal distribution (Figure 1C). Abdominal X-ray demonstrated gas accumulation in the stomach, small intestine, and colon (Figure 1D), resembling ileus. Laboratory data showed a white blood cell (WBC) count of 12,800/mm³ and C-reactive protein (CRP) of 13.9 g/dL, suggesting moderate inflammation. Intravenous fluid therapy and tazobactam/piperacillin hydrate were initiated, and the patient was fasted until the vomiting subsided. 

**Figure 2:**
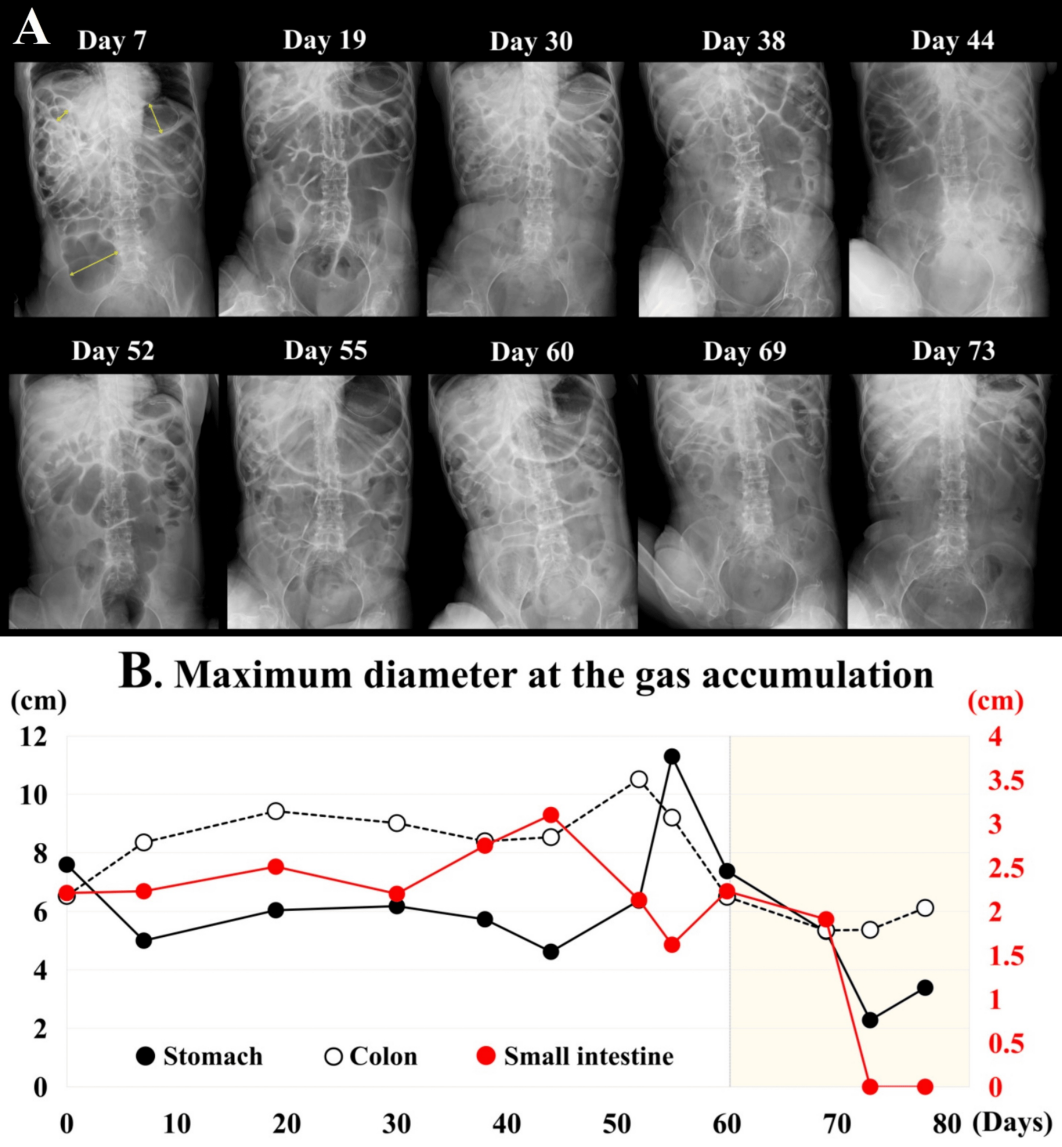
Chronological changes of intestinal gas accumulation (A) Intestinal gas retention on abdominal X-rays Until Day 60, marked gas retention was evident from the stomach to the colon, sometimes resulting in megacolon, with no observed improvement. Once the enteral nutrition conditions were improved through the use of solidifying enteral nutrition and continuous drainage of gastric air via an open nasogastric tube, as described, the gas retention dramatically improved. (B) Measurement of maximum diameters at the gas retention For quantitative analysis, the maximum diameters of the gas accumulation in the stomach, small intestine, and colon were measured using an X-ray system. It is clear that the maximum diameters decreased in the stomach, small intestine, and colon after Day 60, when the procedure was initiated in this case.

The patient recovered from pneumonia steadily with antibiotics, and by Day 7, SpO_2_ had increased to 96%-97% on room air. To decrease the volume of accumulated gas in the digestive tract, mosapride citrate hydrate, dimethicone, pantethine, and lansoprazole were administered through a nasogastric tube (Table 1). Upper and lower endoscopic examinations revealed normal and intact mucosae in the stomach, small intestine, and colon, with no evidence of organic stenosis or obstruction. However, vomiting continued, and a follow-up abdominal X-ray on Day 7 showed persistent gastric and intestinal gas (Figure 2A). Total parenteral nutrition (TPN) was initiated, and vitamin B5 and gastric medications were switched to intravenous administration (Table 1). Vomiting ceased after discontinuation of enteral feedings, and abdominal massage and enemas were performed as needed from Day 10. Prostaglandin (PG) F_2_α was used when bowel sounds were not auscultated to promote expulsion of the colon after Day 12. Although a 12 Fr Salem Sump tube (Muranaka Medical Instruments Co. LTD., Osaka, Japan) had been used for a nasogastric tube since she was at home, it was switched to a 16 Fr Salem Sump tube on Day 21, which was inserted into a length of 60 cm through the nostril for the purpose of allowing the tube tip to reach the gastric pylorus and preventing vomiting. Enteral feeding (1.5 kcal/cc) (Isocal Support 1.5; Nestlé Health Science, Tokyo, Japan) was resumed on Day 18 and was slowly increased to a total of 900 kcal, divided into three meals a day, while the amount of TPN was tapered. The nasogastric tube was occluded for one hour after the end of each feeding to prevent reflux and then left open to allow for passive gastric air release. Involuntary swallowing of saliva was frequently observed, and clonazepam and levodopa-carbidopa hydrate were administered to reduce these involuntary movements (Table 1). On Day 54, her distended abdomen had still not resolved. The nasogastric tube was switched to a double elementary diet (W-ED) tube (16 Fr) (Cardinal Health Inc., Dublin, OH, USA) to evacuate gastric gas while enteral nutrition was administered, although the inner diameter is much narrower than that of the Salem Sump tube. The patient vomited on Days 58 and 59, so the nasogastric tube was returned to a Salem Sump tube (16 Fr) on Day 60. Despite the above interventions, a large amount of intestinal gas continued to accumulate, as shown in Figure 2A.

**Table 1:** Chronological description of medications and procedures PG, Prostaglandin; W-ED, Double elementary diet

Oral medicine	Days										
Mosapride citrate hydrate	0~82										
Dimeticone	0~82										
Sodium valproate	0~10										
Pantethine	0~7			50~82							
Lansoprazole	0~7			50~82							
Daikenchuto	5~82										
Clonazepam	18~82										
Levodopa carbidopa hydrate	18~68										
Intravenous medicine											
Tazobactam/piperacillin hydrate	0~7										
Panthenol	8~49										
Famotidine	8~49										
PG-F2a	12~69 (used when needed)										
Parenteral nutrition											
Peripheral parenteral nutrition	0~7			58~63							
Intravenous hyperalimentation	8~50										
Enteral nutrition											
Isocal Support 1.5	20~58										
HINEX E-gel	63~82										
Nasogastric tube											
Salem Sump tube	0~21 (12 Fr, 55 cm)				22~53 (16 Fr, 60 cm)				60~ (16 Fr, 45 cm)		
W-ED tube 16 Fr, 55 cm						54~59					
Signs and symptoms											
Vomit	Vomit							Vomit			
Abdominal distension	0~64+						65~68±				69~-

**Figure 3:**
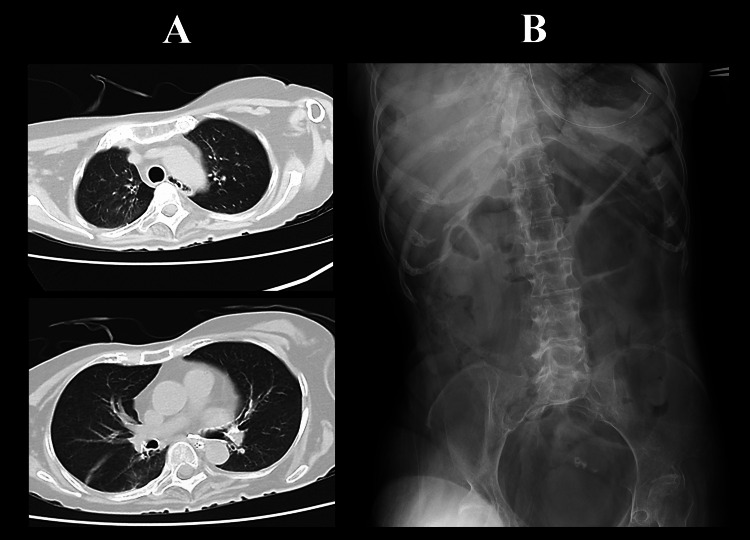
Chest CT and abdominal X-ray at discharge (A) Chest CT taken on Day 78 shows resolution of pneumonia, and (B) abdominal X-ray on the same day demonstrates no recurrence of gas retention in the stomach or intestine. CT, Computed tomography

Consequently, further modifications focused on reducing gastric air. The tube insertion length was shortened to 45-50 cm from the nostril to position it within the gastric cardia, expecting an increased amount of gas expulsion. To reduce empty space within the stomach, enteral nutrition that solidifies in the stomach was used (0.8 kcal/cc) (HINEX E-gel; Otsuka Pharmaceutical Factory, Inc., Naruto, Japan). Furthermore, the frequency of enteral feedings was reduced to twice daily. Following these adjustments, abdominal distension gradually improved, and an abdominal X-ray on Days 69 and 73 demonstrated a remarkable decrease in intestinal gas (Figure 2A). The maximum diameters of the stomach, small intestine, and colon, dilated by gas retention, were measured chronologically, revealing a marked decrease after Day 60 (Figure 2B). By Day 78, the WBC count had normalized to 4,900/mm^3^, CRP had decreased to 0.32 g/dL, pneumonia had improved on chest CT, and abdominal X-ray showed no recurrence of gas accumulation (Figure 3). The patient was discharged home on Day 82.

## Discussion

Despite our patient’s severe intellectual disability since childhood, normal swallowing mechanics were preserved on endoscopic evaluation. However, voluntary swallowing was difficult, leading to involuntary saliva and air swallowing, known as aerophagia [1-3]. Furthermore, the patient was unable to reposition herself, and both lower limbs were stiffened in a flexed position, making the passing of intestinal gas difficult and contributing to intestinal pseudo-obstruction in this case.

Management strategies for intestinal pseudo-obstruction caused by aerophagia include: (1) reducing swallowed air, (2) draining stored air in the stomach, and (3) passing intestinal gas promptly. Improving air swallowing behavior through training can be difficult in patients with severe cerebral impairment. Based on the report mentioning that clonazepam was effective for pathological aerophagia in children with intellectual disabilities [3], clonazepam and antiparkinsonian drugs were administered in our case to decrease the frequency of salivation and involuntary swallowing. However, these medications failed to reduce the amount of gastric and intestinal air volume on X-rays. Abdominal massage and prokinetics were attempted to promote the expulsion of intestinal gas but provided only intermittent symptomatic relief. The authors found that the most important and effective procedure to prevent intestinal pseudo-obstruction in our case was to drain the air from the stomach before it passed the gastric pylorus. Our method utilized multiple tactics simultaneously, including: (1) use of enteral nutrition that immediately solidifies in the stomach, along with shortening the time of tube occlusion to prevent reflux; (2) reducing feeding frequency to twice daily; (3) inserting a nasogastric tube so that the tip of the tube is placed at a depth of 5-10 cm from the gastric cardia; and (4) placement of a 16 Fr nasogastric tube that was kept open at all times outside of feedings to allow for passive gas release. Although the frequency of swallowing did not decrease in this case, these procedures dramatically reduced gastric and intestinal gas volume, alleviating the abdominal distention. The method in this case is not difficult and is safe to perform at home. The use of enteral nutrition, which solidifies in the stomach, not only prevents vomiting but also watery diarrhea. Such a type of enteral feeding would allow a nasogastric tube to be kept open for 24 hours. One recommendation is that, as the length of the nasogastric tube to be inserted depends on the individual's height and posture - especially in a pediatric patient - it is safer to confirm by X-ray that the tip of the tube has been placed in the right location to allow the gastric air to be expelled.

Aerophagia is a chronic condition that is difficult to correct in patients with severe intellectual disabilities, and medical intervention is often unnecessary in asymptomatic individuals [9]. However, patients who are unable to complain of nausea or abdominal pain are at high risk of severe illness when symptoms arise. A report of intestinal perforation caused by pathological aerophagia highlights the importance of close monitoring in these cases [10]. Although there are limitations to the conclusions in this case - due to the single adult case, the inability to directly measure swallowing frequency, and potential bias in attributing outcomes to specific interventions - the authors believe this to be an effective method of evacuating air from the stomach regardless of cause and that it can be tried in homecare patients on enteral nutrition who show massive intestinal gas retention.

## Conclusions

Patients with severe intellectual disability, managed with a nasogastric tube, can present with aerophagia and should be carefully observed for excessive abdominal distention. In such cases, procedures to drain air from the stomach may be more effective in preventing recurrence than efforts to expel intestinal gas.
